# Partial loss of actin nucleator actin‐related protein 2/3 activity triggers blebbing in primary T lymphocytes

**DOI:** 10.1111/imcb.12304

**Published:** 2019-12-23

**Authors:** Peyman Obeidy, Lining A Ju, Stefan H Oehlers, Nursafwana S Zulkhernain, Quintin Lee, Jorge L Galeano Niño, Rain YQ Kwan, Shweta Tikoo, Lois L Cavanagh, Paulus Mrass, Adam JL Cook, Shaun P Jackson, Maté Biro, Ben Roediger, Michael Sixt, Wolfgang Weninger

**Affiliations:** ^1^ Immune Imaging Program The Centenary Institute Faculty of Medicine and Health The University of Sydney Camperdown NSW 2050 Australia; ^2^ School of Biomedical Engineering The University of Sydney Sydney NSW 2006 Australia; ^3^ Heart Research Institute and Charles Perkins Centre The University of Sydney Sydney NSW 2006 Australia; ^4^ Tuberculosis Research Program The Centenary Institute The University of Sydney Camperdown NSW 2050 Australia; ^5^ Discipline of Infectious Diseases & Immunology Marie Bashir Institute The University of Sydney Sydney NSW 2006 Australia; ^6^ Sydney Medical School The University of Sydney Sydney NSW 2006 Australia; ^7^ EMBL Australia Node in Single Molecule Science School of Medical Sciences the ARC Centre of Excellence in Advanced Molecular Imaging University of New South Wales Kensington NSW 2033 Australia; ^8^ Department of Molecular Genetics and Microbiology University of New Mexico School of Medicine Albuquerque NM 87131 USA; ^9^ Central Clinical School Sydney Medical School The University of Sydney Sydney NSW 2006 Australia; ^10^ Institute of Science and Technology Klosterneuburg 3400 Austria; ^11^ Department of Dermatology Royal Prince Alfred Hospital Camperdown NSW 2050 Australia; ^12^ Discipline of Dermatology Sydney Medical School The University of Sydney Sydney NSW 2006 Australia; ^13^ Department of Dermatology Medical University of Vienna Vienna 1090 Austria

**Keywords:** actin cytoskeleton, Arp2/3, blebbing motility, cellular morphology, primary CD8^+^ T cell

## Abstract

T lymphocytes utilize amoeboid migration to navigate effectively within complex microenvironments. The precise rearrangement of the actin cytoskeleton required for cellular forward propulsion is mediated by actin regulators, including the actin‐related protein 2/3 (Arp2/3) complex, a macromolecular machine that nucleates branched actin filaments at the leading edge. The consequences of modulating Arp2/3 activity on the biophysical properties of the actomyosin cortex and downstream T cell function are incompletely understood. We report that even a moderate decrease of Arp3 levels in T cells profoundly affects actin cortex integrity. Reduction in total F‐actin content leads to reduced cortical tension and disrupted lamellipodia formation. Instead, in Arp3‐knockdown cells, the motility mode is dominated by blebbing migration characterized by transient, balloon‐like protrusions at the leading edge. Although this migration mode seems to be compatible with interstitial migration in three‐dimensional environments, diminished locomotion kinetics and impaired cytotoxicity interfere with optimal T cell function. These findings define the importance of finely tuned, Arp2/3‐dependent mechanophysical membrane integrity in cytotoxic effector T lymphocyte activities.

## Graphical abstract

Quantification of cytotoxic effector T lymphocytes (CTLs) morphology using standard biomedical assays and developed image analysis algorithms showed that the reduction of actin‐related protein (Arp)3 levels impaired F‐actin content maintenance, leading to defects in CTL functionality. The knockdown CTL motility mode, instead of the actin‐rich lamellipodia‐based migratory strategy, displays blebbing‐like migration at the cost of reduced migration speed. Thus, the role of the Arp2/3 complex is not redundant among the various mechanochemical coordinators involved in the leading‐edge formation in CTLs.

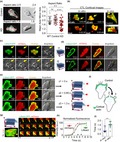

## Introduction

Cytotoxic effector T lymphocytes (CTLs) provide immunosurveillance against invading pathogens and malignant cells.^1^, ^2^ To effectively contribute to successful immune responses, CTLs employ flexible migratory programs that are informed by extrinsic (e.g. chemokines, stromal elements) and intrinsic (signaling molecules, cytoskeleton) factors.^3^, ^4^, ^5^ Thus, migrating CTLs *in situ* adopt a polarized cell shape defined by the formation of a lamellipodium at the leading edge and a uropod at the rear of the cell.^6^ This ameboid migration mode is typical of leukocytes, including granulocytes and lymphocytes, and is thought to facilitate the rapid movement of these cells to and within sites of inflammation and infection.^7^, ^8^

The structure and function of the actomyosin cortex is highly cell‐type dependent and governed by the submembranous cytoskeleton, which comprises actin network filaments, actin‐binding proteins and myosin‐II.^9^ Together, these actin regulators control the cell shape changes requisite for cell migration through the interstitial spaces of organs. As lymphocytes have unique migratory demands, it is important to understand the biomechanics of the actomyosin cortex, the precise contribution of its regulators and the consequences of its disturbance upon lymphocyte migration and effector functions.

At the molecular level, precise T cell migration is associated with constant remodeling of the cytoskeleton, particularly at the leading edge, which provides the engine that propels the cell membrane forward. Remodeling of the lamellipodium is facilitated by polymerization and branching of actin filaments. These are mediated by actin nucleation factors such as the actin‐related protein 2/3 (Arp2/3) complex.^10^ The Arp2/3 complex is a 225‐kD macromolecular assembly comprising seven subunits: five highly conserved but unique subunits of ARPC (ARPC1–5) and two ARP (Arp2 and Arp3) that structurally mimic actin monomers. For activation, Arp2/3 requires one or more nucleation‐promoting factors including the verprolin‐homologous protein (WAVE) family, the Wiskott–Aldrich syndrome protein family and the hematopoietic lineage cell‐specific protein 1 from the cortactin family.^11^ When activated, the Arp2/3 complex binds to the side of a pre‐existing actin filament, and Arp2 and Arp3, together with an additional actin monomer, form a nucleation core. This trimer then operates as a template for daughter filament elongation.^12^, ^13^ Functionally, the Arp2/3 complex is critical for cell polarity, cell migration^14^ and cellular cortex network integrity.^15^ Other studies have revealed the Arp2/3 complex as a critical mediator of cytokinesis in multiple cell types.^16^, ^17^, ^18^ In Arp3‐KD human natural killer cells, the assembly and maturation of the lytic synapse were impaired while the integrin and natural killer receptor signaling were unaffected.^11^ The disruption of Arp2 or Arp3 in the Arp2/3 complex often leads to a decrease in the expression of other Arp2/3 complex components hindering the integrity of the complex resulting in severe phenotypes such as disrupted T cell receptor (TCR) expression.^19^ Moreover, ARPC4 knockdown in the epidermis leads to psoriasis‐like skin complications,^20^ and global Arp2 mutations are lethal in *Dictyostelium*.^21^ ARPC3 depletion results in embryonic lethality in mouse.^22^ In humans, *ARPC1B* mutations result in symptoms of immune dysregulation including mild bleeding tendency.^23^ Furthermore, a recent study by Schaffer *et al*. highlighted the importance of Arp2/3 regulation in a human disorder known as pachygyria, where a mutation in CTNNA2 leads to an overactivity in Arp2/3, resulting in disordered cortical neuronal migration.^24^ Silencing Arp2 and Arp3 in Jurkat T cells results in failure to spread on anti‐CD3‐coated coverslips, switches the F‐actin‐rich lamellipodia leading edge to polarized filopodia‐like structures, which established the link between the β2‐integrin activation and functional Arp2/3.^19^ Silencing these genes also impaired immunological synapse (IS) formation in ARPC2‐knockout T cells *in vitro*.^25^ A recent study showed that conditional knockout of ARPC2 in T‐cells results in decreased expression of the TCR and impaired T‐cell homeostasis.^25^ Although the role of Arp2/3 in lamellipodia formation has been studied intensively, its roles in T lymphocytes and their immunological function remain to be completely defined. Moreover, the consequences of disrupted Arp2/3 complex, particularly the Arp3 subunit, on T‐cell locomotion and morphology *in vivo* are neither characterized nor quantified.

The actomyosin cortex is usually observed immediately adjacent to the cell membrane. However, under certain circumstances, the cell membrane transiently detaches from the actin cortex resulting in the formation of blebs.^26^ Blebs have long been observed under physiological circumstances such as during cell death (apoptosis) and cytokinesis (at the poles of dividing cells), particularly in embryonic cells (where the blebs are known as lobopodia).^27^, ^28^ Currently, the center of speculation is on the factors that facilitate membrane detachments such as reduced actin polymerization or reduced cortical contractility.

As an emerging concept, blebbing is also considered a motility mode occurring under certain conditions during cell migration in two‐dimensional (2D) and 3D microenvironments (reviewed in Blaser *et al*.^26^ and Paluch and Raz^29^). For example, in Walker carcinoma cells, this mode represents a putative escape mechanism particularity observed during protease‐inhibitor treatment.^30^ The zebrafish primordial germ cells also use blebbing migration toward distant targets in the gonad.^26^ Blebbing has also been observed in other cell lines such as neutrophil‐like cells in microfluidics devices,^31^ when adhesion to the substrate is reduced in cells from patients with leukocyte adhesion deficiency.^32^ Fibroblasts following cell cortex ablation^33^ or epithelial cells in which Arp2/3 activity was hindered with an Arp3 short hairpin RNA (shRNA) or CK‐689 will also bleb.^34^ These findings suggest that stimuli leading to blebbing motility are cell type specific. Thus, further studies that focus on the molecular mechanism allowing the switch to blebbing migration are necessary to understand processes leading to the formation of different protrusions.

Nevertheless, T cells are thought to exclusively utilize lamellipodia‐ or filopodia‐based locomotion, even when migrating using an integrin‐independent strategy.^35^ Unlike blebbing, lamellipodia motility seems to allow precise sensing of the microenvironment and might provide navigation guidance based on the rigidity of the substrate measured via formation of focal adhesions.^36^ These require precise regulation of actin polymerization and dendritic network growth and also matrix proteolysis. Although the architecture of lamellipodial actin filaments and mechanical properties of cortical actin are critical for active migration of T cells, relatively little is known about how the cytoskeleton network and different subunits of Arp2/3 regulate the movement, morphology and function of T cells in 3D environments.

To gain insight into the mechanisms and the role of the Arp3 subunit in morphology and function of primary T cells, we used shRNA mediated Arp3 knockdown (KD) to study the role of this protein in controlling cortical actin organization. We have also developed image analysis algorithms to quantify Arp3‐KD effects on cell shape. Our results demonstrate that CTLs with compromised Arp3 levels exhibit impaired F‐actin content maintenance leading to defects in CTL functionality. We further show that the CTL motility mode, instead of the actin‐rich lamellipodia‐based migratory strategy, displays blebbing‐like migration at the cost of reduced migration speed. Taken together, our results suggest that optimal mechanophysical and biochemical properties of the actomyosin cortex, as maintained by the Arp2/3 complex, are essential for the proper functioning and effective migration of CTLs. While many key issues still need to be addressed, our study provides a model system for studying the molecular and physiological aspects of blebbing migration.

## Results

### Reduction of Arp3 in cytotoxic T cells reduces total F‐actin content

**Figure 1:**
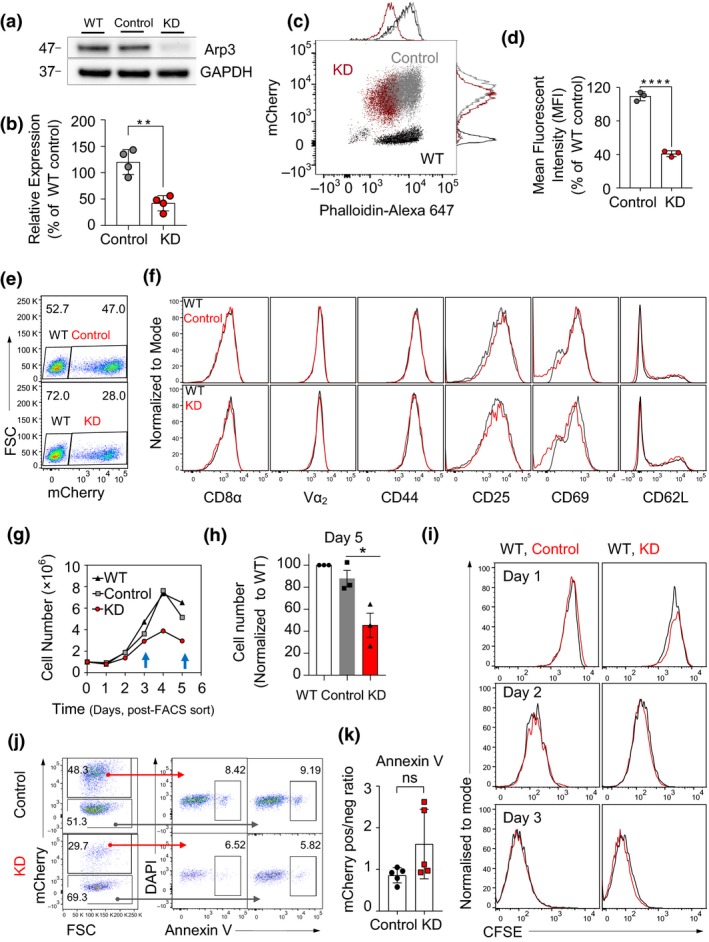
Evaluation of the effects of Arp3‐knockdown (KD) on F‐actin, proliferation, cytotoxic phenotype and apoptotic status. **(a)** Representative immunoblot of Arp3 (*ACTR3*) expression levels in cells transduced with anti‐Actr3 short hairpin RNA (shRNA), control shRNA and non‐transduced wild‐type (WT) cytotoxic effector T lymphocytes (CTLs) lysed at day 5 post‐harvest. See also Supplementary figure 1a. **(b)** The column graph shows the densitometry analysis of the Arp3 protein band. Arp3 expression signal was normalized against WT expression level and corrected against glyceraldehyde 3‐phosphate dehydrogenase (GAPDH) signal (*n* = 4 independent experiments, **P = 0.0014, also see Figure 1a, b). **(c, d)** Total F‐actin content in the Arp3‐KD. **(c)** Flow cytometry plot demonstrating phalloidin staining of F‐actin; KD in red, WT in black, control in gray. **(d)** Quantification of total F‐actin in Arp3‐KD CTLs on day 7 post‐isolation, 48 h post‐fluorescence activated cell sorting (*n* = 3 independent experiments, ****P < 0.0001). The relative fluorescence expressions were normalized to WT control. **(e)** A representative FACS plot shows transduction efficiency of splenocytes harvested from Lifeact‐GFP×OT‐I mice on day 5 post‐transduction (*n* = 4 independent experiments). **(f)** FACS histograms of transduced T cells (red) and WT [non‐transduced cells (gray) acting as an internal control] stained with anti‐CD8α, anti‐Vα_2_, anti‐CD44, anti‐CD25, anti‐CD69 and anti‐CD62L antibodies as indicated (data from day 6 following T cell isolation; representative results of three independent experiments). **(g)** Growth curve of FACS‐sorted transduced CTLs and WT control over 5 days (counting commenced 48 h post‐thawing, day 6 post‐harvest). See also Supplementary figure 1c, d. **(h)** Quantification of cell numbers compared with WT cells on day 5 post‐FACS sort (*n* = 3 independent experiments, *P = 0.03). **(i)** Carboxyfluorescein diacetate succinimidyl ester (CFSE) profiles of control transduced and Arp3‐KD CTLs for 3 days post‐staining on days 6–9 post‐harvest (representative of three independent experiments between days 6 and 11 post‐harvest, see also Supplementary figure 1b for CFSE labeled prior to cytokine stimulation of splenocytes). **(j)** Representative flow cytometry plots illustrating Annexin V staining in vector control, Arp3‐KD and non‐transduced internal control. **(k)** The mCherry expression ratio between transduced and control (non‐transduced) Annexin V^+^ cells (data pooled from two independent experiments; days 6–8 post‐harvest). ns denotes not significant. Data are represented as mean ± s.d.; statistical significance was calculated between control and KD cells using the unpaired Student's *t*‐test. DAPI, 4,6‐diamidino‐2‐phenylindole; FSC, forward scatter; GFP, green fluorescent protein.

To explore the consequences of modulated Arp3 expression levels in CTLs, we employed a retroviral knockdown strategy. Activated CTLs were transduced with viral particles encoding an shRNA against *Actr3* (encoding Arp3) or a nonsilencing shRNA (control). This RNA interference technique was used as, compared with other approaches such as small interfering RNA, it offers sustainable KD and less off‐target effects.^37^ The construct also contained mCherry to facilitate the identification of transduced T cells. CTLs were generated from naïve T cells isolated from OT‐I TCR transgenic mice in which the CD8^+^ T cells express a TCR specific for the SIINFEKL peptide of ovalbumin presented on k^b^. Mice were crossed to either recombination activating‐gene 1 (*Rag1* ^–/–^) background or enhanced green fluorescent protein (EGFP)‐Lifeact mice, in which EGFP is fused to the F‐actin‐binding peptide, Lifeact.^38^ Molecular characterization of fluorescence‐activated cell sorting (FACS)‐sorted mCherry^+^ CTL was used to evaluate the efficacy of shRNA‐mediated Arp3 KD. This indicated that introduction of shRNA‐Actr3, but not control shRNA, led to reduced expression but not complete ablation of the endogenous Arp3 subunit (Figure 1a, b and Supplementary figure 1a). Flow cytometry analysis of phalloidin‐stained CTLs, a readout of filamentous actin, indicated that total F‐actin content in shRNA‐Actr3‐transduced T cells was reduced by approximately 60% (Figure 1c, d). This is in line with a critical role played by Arp3 subunit in the Arp2/3 complex formation, function^39^ and maintenance of F‐actin levels in CTLs.

### T cell phenotype remains unaltered after Arp3 knockdown

The actin cytoskeleton provides the framework to maintain T cell membrane organization dynamics.^40^ Thus, we examined whether Arp3 knockdown resulted in changes in the surface expression of the TCR or activation markers, including CD44, CD25, CD69 and CD62L, in activated CTLs (Figure 1e, f). Despite the reduction in the total F‐actin content in CTLs following Arp3 knockdown, the expression profile of examined molecules was comparable to CTLs transduced with control shRNA. Experiments in this study relied on the *in vitro* expansion, transduction and sorting of mCherry^+^ OT‐I cells. In these experiments, we noticed a reduction in the number of Arp3‐KD cells maintained in culture, particularly at day 5 following cell sorting (Figure 1g, h). The relative loss of Cherry^+^ cells in the Arp3‐KD population correlated with the reduction in the total cell number in the plate. This suggests that relative loss of Cherry^+^ cells is not a result of the loss of the KD construct. Therefore, we next evaluated CTL proliferation by carboxyfluorescein diacetate succinimidyl ester (CFSE) and CellTrace violet dilution. Regardless of the virus employed for transduction or the transduction efficiency, all CTLs examined exhibited identical proliferation profiles (Figure 1i, Supplementary figure 1b). Nevertheless, the contribution of mCherry‐negative (non‐transduced) CTLs increasedrp3‐KD group, in contrast to controls (Supplementary figure 1c, d), suggesting a survival deficit in Arp3‐KD CTLs. To test whether this was a result of increased rates of apoptosis, we assessed the apoptotic status of transduced cells using Annexin V staining, which binds to the phosphatidylserine on the outer leaflet of the plasma membrane in the early stages of apoptosis. The level of Annexin V binding varied between 5 and 10% but was not enriched in transduced *versus* non‐transduced cells for either Arp3 knockdown or control (Figure 1j, k). Therefore, apoptotic cell death was not different at any time point (days 4–7) where cells were used for functional assays. Collectively, these findings suggest that, while the reduction of Arp3 showed no significant effect on the examined cell cytotoxic surface markers and rates of proliferation during days 2–11 post‐CTL harvest. Thus, the Arp2/3 complex is potentially important for CTL survival.

### *In vivo* survival of adoptively transferred Arp3‐KD OT‐I CTLs critically depends on Arp3 expression

**Figure 2:**
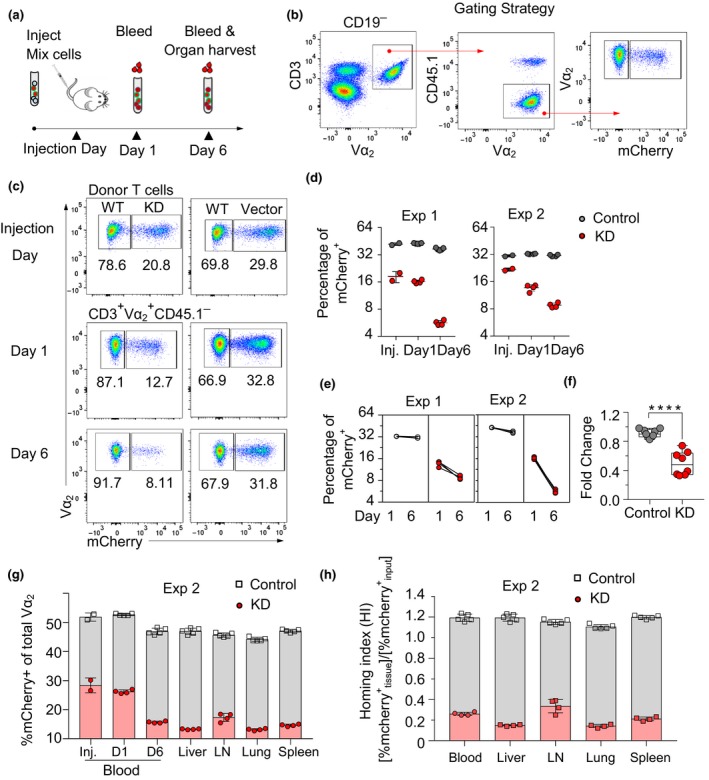
*In vivo* survival of adoptively transferred Arp3‐KD OT‐I cytotoxic effector T lymphocytes (CTLs) critically depends on Arp3 expression. **(a)** Schematic representation depicting the tracing of adoptively transferred transduced OT‐I cells and non‐transduced wild‐type (WT) OT‐I cells (a total of 20 × 10^6^) injected into B6.SJL/*Ptprc* ^*a*^ (CD45.1) mice. Cells were evaluated by flow cytometry on injection day (day 6 post‐harvest *in vitro*) and subsequently on day 1 and day 6 post‐injection of cells. **(b)** Gating strategy used in flow cytometry analysis to detect the percentage of the mCherry^+^ donor CTLs in recipient mice. **(c)** Representative fluorescence‐activated cell sorting (FACS) plot of transduction efficiency on day 0 (day 6 post‐harvest) prior to adoptively transferring CTLs (top), day 1 (middle) and day 6 (bottom) post‐injection. **(d)** The percentage of mCherry^+^ cells in Actr3‐KD and control CTLs on days 0, 1 and 6 post‐injection [two independent experiments, experiment 1 (Exp 1, *n* = 4) and Exp 2, KD *n* = 4, control *n* = 5 mice/group]. **(e)** Paired analysis of mCherry^+^ percentages normalized against percentages detected in blood on day 6 post‐injection. **(f)** Boxes and Whisker graph showing pooled data from two independent experiments (KD *n* = 8 mice, control *n* = 9 mice). **(g)** Bar graphs show the percentage of mCherry^+^ of total Vα_2_ on injection day, day 1 and day 6 in control CTLs in blood and other organs, respectively. **(h)** The percentage of mCherry^+^ cells for each tissue was normalized by the input percentage of mCherry^+^ cells to obtain the Homing Index. See also Supplementary figure 1. Panel f, statistical analysis was performed with the Mann–Whitney *U* test. ****P < 0.0001. KD, knockdown; LN, lymph node.

To characterize the effect of Arp3 knockdown on T cell homing and survival *in vivo*, we adoptively transferred CD45.2^+^ Arp3‐KD or control CTLs 5 days post‐transduction into CD45.1^+^ *Ptprc* ^*a*^ congenic recipient mice. To evaluate cell survival over time, mice were subsequently bled on day 1 and day 6 post‐adoptive cell transfer, together with organ harvest on day 6 (Figure 2a). Donor CTLs were discriminated on the basis of CD45.1 expression by flow cytometry (Figure 2b). Similar to the *in vitro* data, the fraction of Arp3‐KD T cells in blood declined with time, relative to the fraction of non‐transduced cells (Figure 2c–f). We also found that Arp3‐KD OT‐I CTLs were capable of entering lungs, spleens, livers and lymph nodes. Arp3 depleted CTLs accumulated preferentially in lymph nodes compared with control cells, but to a lesser extent in lungs, spleens and livers (Figure 2g, Supplementary figure 2a–c). These slight differences in trafficking and distribution of the leukocytes in the body might be related to motility alteration as a result of lack of proper Arp2/3 function in Arp3‐KD CTLs. Together, these findings suggest that Arp3 knockdown impedes the survival of effector CTLs *in vivo* which may be a result of lack of coordination of the cytoskeletal structures, but only has moderate effects on their homing throughout the organism (Figure 2h).

### Arp3 knockdown results in impaired OT‐I CTL migration speed and more confined migration

**Figure 3:**
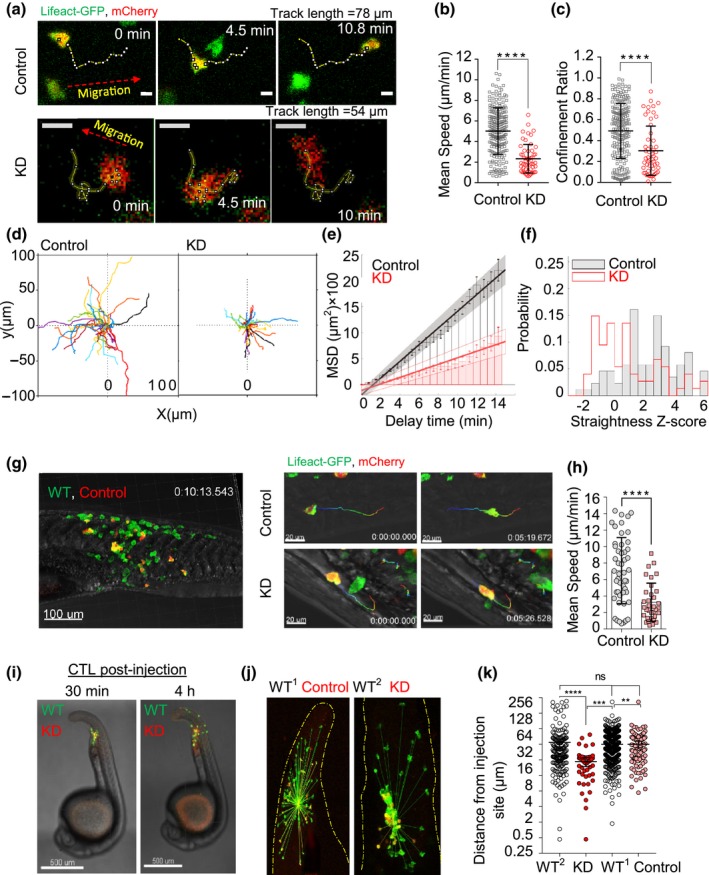
The influence of Arp3‐KD on cytotoxic effector T lymphocyte (CTL) migration *in vitro* and *in vivo*. **(a)** OT‐I × Lifeact‐GFP Arp3‐KD CTLs embedded in collagen and imaged with confocal microscopy for approximately 15 min. Scale bar: 10 μm. **(b** and **c)** Distribution of mean speed and confinement ratios of transduced CTLs on day 5 post‐harvest or day 2 post‐thawing. Data were pooled from three fields of view, from three independent experiments. The values are mean ± s.d. of 220 control and 55 KD cells with the filter being imposed on cell track analysis (excluded track length < 5 μm). **(d)** A representative two‐dimensional reconstruction of single‐cell tracks from one experiment aligned to a common origin (41 and 47 track numbers for control and KD, respectively, see Supplementary videos 1 and 2). **(e)** Bars showing mean square displacements (MSDs) ± s.d. (error bars), linear regression (line) and confidence intervals (shaded areas) with a diffusion coefficient of 26.7 and 10.4 μm^2^ min^−1^ for control and KD cells, respectively. **(f)** Straightness Z‐score. **(g**, left panel**)** Zebrafish embryos microinjected with approximately 60 control‐KD OT‐I × Lifeact‐GFP T cells (WT = green only, control = green and red). **(g**, right panel**)** A snapshot of migration of control and KD cells at 0 and 5 min with the 10‐min rainbow color tracking trajectories. **(h)** Distribution of mean speed of CTLs *in vivo in* about 20 min post‐microinjection. Data were pooled from two (control) and three (KD) independent experiments for 10–20 min imaging (control cells *n* = 49, KD *n* = 34, 2–3 zebrafish/group, 1–2 ROI(s)/embryo, 5–19 cells/image). **(i, j)** A representative image of CTLs at early (30 min) and late (4 h) time points post‐injection (non‐transduced cell serves as internal control, the yellow dashed line indicates the tails part of larva). **(k)** Distribution of distances from injection site, data were pooled from three independent experiments with 2‐ to 4‐h migration time (4–12 zebrafish/group, WT ^1^ *n* = 394, WT ^2^ *n* = 175 internal control mixed with control *n* = 107 and KD *n* = 45, respectively). *In vivo* imaging performed at 37°C on day 6 or 7 post‐harvest. Statistical analyses in **b, c** and **h** were performed using unpaired Student's *t‐*tests and in **k** using one‐way ANOVA, followed by Tukey's multiple comparisons test. ns denotes not significant. Data are represented as mean ± s.d. in **h** and mean± 95% CI in **k**. **P = 0.002, ***P = 0.0003 and ****P < 0.0001. GFP, green fluorescent protein; KD, knockdown; ROI, region of interest.

The requirement of Arp2/3 for *in vivo* survival of CTLs prompted us to quantify the precise 3D migration characteristics of Arp3 depletion on CTL locomotion in 3D environments. First, we visualized OT‐I or Lifeact‐GFP CTLs transduced with Arp3‐shRNA or control shRNA embedded within the collagen matrix *in vitro* by confocal microscopy. Arp3‐KD CTLs exhibited an arrested phenotype compared with control CTLs (Figure 3a). This was reflected by a significant reduction in the average migratory speed (from 5 ± 2.3 to 2.3 ± 1.4 μm min^−1^, Figure 3b). The confinement ratio, reflecting the degree of exploratory behavior of the cells, was also significantly lower in the knockdown population with shorter track lengths (Figure 3c, d). In control conditions, cells show both straight and meandering migration, whereas, after Arp3‐KD, most cells show meandering migration for most of the time Supplementary videos (1 and 2). We also observed that the Arp3‐KD cells had on average a lower diffusion coefficient (10.4 μm^2^ min^−1^) than the control cells (26.7 μm^2^ min^−1^, Figure 3e). While both control and KD cells had a linear relationship in time *versus* mean square displacement (MSD), further experiments demonstrated that there was a difference in the “confined” and “straight” plots between the two groups. When compared with the control, Arp3‐KD CTLs possessed a more negative score, suggesting that their migratory behavior was more “confined” (Figure 3f).

Next, to understand the effect of Arp3 knockdown on CTL migration *in vivo*, we utilized a zebrafish model. Zebrafish larvae are transparent and have gained popularity for studying cell trafficking in real‐time *in vivo*.^41^, ^42^ Furthermore, the lack of an adaptive immune system during the first 2 weeks of the zebrafish embryonic life enables xenotransplantation of T cells with no adverse effects.^43^ We, therefore, microinjected transduced CTLs into zebrafish embryos to track their migratory behavior *in vivo*. We observed a similar migratory impediment in CTLs after Arp3 knockdown compared with control T cells (mean track speed of 3.3 ± 2.5 *versus* 7 ± 4 μm min^−1^, Figure 3g, h). When zebrafish embryos were imaged 2‐ and 4‐h post‐injection, the distance traveled by Arp3‐KD CTLs *in vivo* was approximately 66% shorter than that observed in control cells (Figure 3i–k). Collectively, these data demonstrate that a defect in the Arp3 subunit of the Arp2/3 complex leads to a significant reduction in the migratory capacity of CTLs both *in vitro* and *in vivo*.

### Downregulation of Arp3 affects morphology and membrane dynamics of cytotoxic T cells

**Figure 4:**
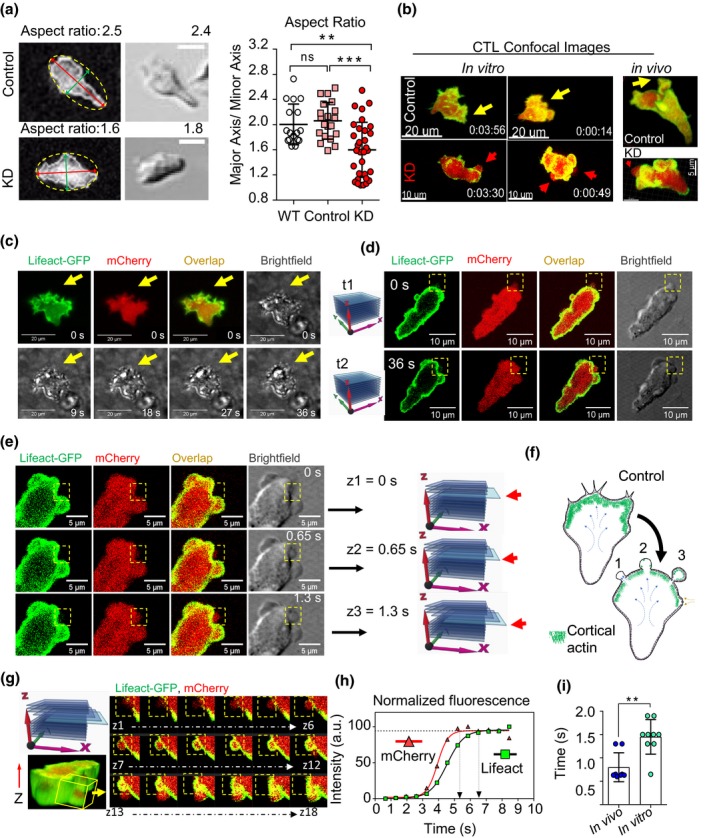
The Actr3‐KD T cells become rounded and switch to blebbing migration mode. **(a)** The aspect ratio [maximum (red line) minimum (green line) length] measured using ellipse fitting (yellow dashed line) into the binary mask of Actr3‐KD and control cells at the bottom of 96‐well plates. The graph (right) shows mean ± s.d. pooled from two control (*n* = 20 cells), two wild‐type (WT;* n* = 17) and three KD (*n* = 32) independent experiments; one‐way ANOVA, followed by Tukey's multiple comparisons test was performed, **P = 0.0017, ***P = 0.0001. Bars, 10 μm. **(b)** Representative images of control cells with lamellipodia at the leading edge (yellow arrows) and KD cytotoxic effector T lymphocytes (CTLs) with balloon‐like protrusions at the leading edge (red arrows) in three‐dimensional collagen matrices and *in vivo* zebrafish model. **(c)** Fluorescence microscopy images of control cells at *t* = 0 s with brightfield images at *t* = 0–36 s and spiky lamellipodia at leading edge (yellow arrows). **(d)** Observation of individual blebs (yellow boxes) of Arp3‐KD CTLs in different z‐stacks, traceable in brightfield images of Arp3‐KD CTLs (yellow boxes). **(e)** Visualization of bleb formation using consecutive frames in one z‐stack (yellow boxes). The time interval was calculated by dividing the time used in capturing the entire z‐stack by the number of frames. z1 = 0 s expansion initiation, z3 = 1.3 s expanded bleb devoid of actin. **(f)** Schematic of switching from lamellipodia formation in control to blebbing in Arp3‐KD CTLs (life cycle of a bleb: one devoid actin, two recruiting actin and three filled with actin). **(g)** A representative time series from 18 consecutive frames in a z‐stack of an Arp3‐KD CTLs embedded in collagen on day 5 post‐harvest is displayed. **(h)** Quantification and **(i)** distribution of Lifeact‐GFP (green) recruitment relative to the cell membrane (red, cytoplasmic mCherry) into the bleb membrane protrusions in Arp3‐KD CTLs *in vivo* and *in vitro*. Data were pooled from two independent experiments (represented as mean ± s.d.), cell number, *in vivo n* = 8 blebs (four cells, in two zebrafish embryos), *in vitro n* = 9 blebs (four cells), and an unpaired Student's *t*‐test was performed, **P = 0.0014. GFP, green fluorescent protein; KD, knockdown.

Given the impediment in the migration of Arp3‐KD CTLs, we sought to better understand the mechanistic consequences of Arp3 deficiency in CTL migration by monitoring individual cells. Motility, an essential feature of T cell function, is dictated by cell polarity. Polarized cells, exhibiting characteristic lamellipodia and uropod, are migratory while rounded cells are not.^44^ To decipher the effects of Arp3 knockdown on cell morphology, CTLs were imaged in tissue culture plates coated with a thin layer of type I collagen. We found that Arp3‐KD CTLs displayed a more rounded morphology compared with the polarized morphology in control and non‐transduced CTLs (Figure 4a). Further analysis of cell morphologies revealed that, as expected, control CTLs exhibited features reminiscent of lamellipodia and filopodia throughout their migration (Figure 4b, left and right top panels and Figure 4c). Surprisingly, however, we consistently observed that a reduction in Arp3 levels resulted in spherical protrusions on the cell surface initially devoid of actin in CTLs, as visualized by the absence of Lifeact‐GFP signal in both T cells migrating *in vitro* and *in vivo* (Figure 4b, bottom left and right panels). Absence of actin filaments identified these structures as membrane blebs (Figure 4d, observation in different z‐stack). We then analyzed, in‐depth, the dynamics of actin recruitment into each bleb by 3D real‐time imaging in transduced CTLs (Figure 4e, observation in one z‐stack). By addressing the temporal order of membrane protrusion and actin recruitment to each bleb, we were able to demonstrate that membrane protrusion occurred prior to F‐actin recruitment to each bleb (Figure 4f). The average time difference in the detection of Lifeact‐GFP (F‐actin) relative to cell membrane indicated by mCherry was about 1 s (Figure 4g–i).

**Figure 5:**
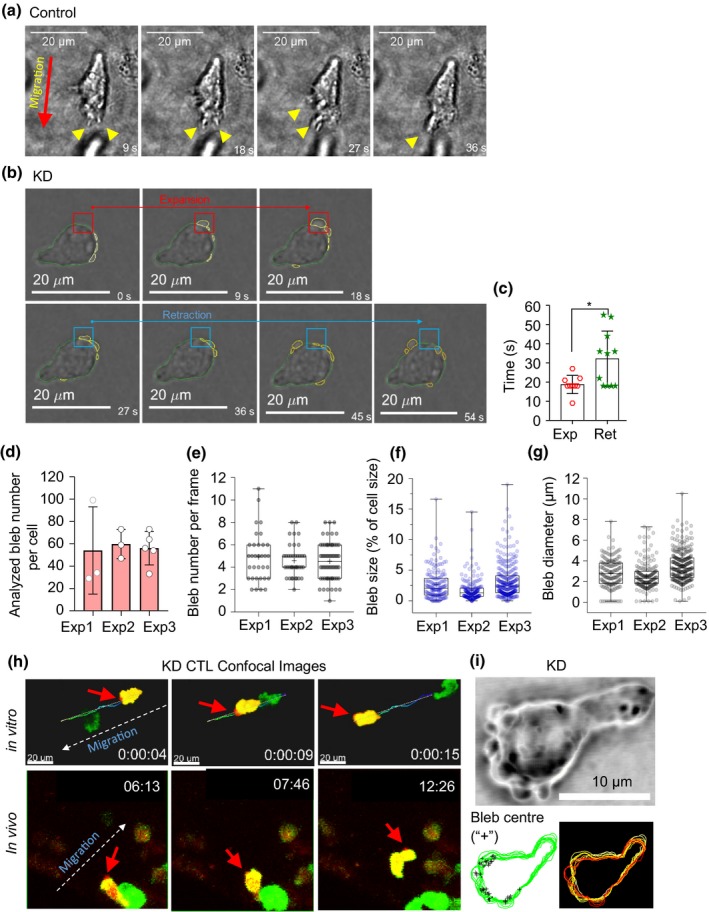
Properties of blebs in Arp3‐KD cytotoxic effector T lymphocytes (CTLs). **(a)** Representative (Lifeact‐GFP) brightfield image of the control cell displaying lamellipodia at the leading edge (yellow arrowheads). **(b)** Images of Arp3‐KD CTL blebbing during an expansion (red boxes) and retraction (blue boxes) were analyzed by manually tracking the bleb masks in consecutive frames. **(c)** Distribution of bleb expansions and retractations in Arp3‐KD CTLs embedded in three‐dimensional collagen (*n* = 3 independent experiment); nine expanding and ten retracting blebs in three or four cells/experiment on day 6 post‐harvest (*P = 0.016, an unpaired Student's *t*‐test was used, and data are represented as mean ± s.d.). **(d)** Distribution of the total number of observed and analyzed blebs per cell (cell number: Exp1 = 3, Exp2 = 3, Exp3 = 5, imaging for 2–3 min on days 5–7 post‐harvest). Data are represented as mean ± s.d. **(e)** Distribution of the observed bleb numbers in each frame (number of blebs Exp1 = 33, Exp2 = 39, Exp3 = 64). **(f)** Distribution of the size of the observed bleb (area) normalized to the size (area) of the whole‐cell expressed as bleb size percentage of cell size (number of blebs: Exp1 = 162, Exp2 = 179, Exp3 = 290). **(g)** Distribution of the observed bleb, longest diameter expressed in micrometer (number of blebs: Exp1 = 162, Exp2 = 179, Exp3 = 290). See also Supplementary table 1. **(h)** Blebbing frequently observed at the leading edge of cells migrating in three‐dimensional collagen matrices *in vitro* (top panels) and *in vivo* (bottom panels) in zebrafish embryos. White arrows show migration direction, red arrows depict the bleb locations. **(i)** Representative images displaying blebs at the leading edge (top panel), the overlap of each bleb center (+) in time (left bottom panel) and overlap of time‐lapse images of cell border (right bottom panel, white is time 0 and dark red is the last time point). **(e–g)** The results from three independent experiments represented in a box and Whisker plot format, with a median, first and third quartiles outlined by the box, and minimum and maximum values of the data set denoted by Whiskers. GFP, green fluorescent protein; KD, knockdown.

Finally, we measured the size and the number of blebs per imaging frame (Figure 5) using brightfield imaging as time‐lapse imaging resulted in photobleaching of the mCherry fluorophore. Blebs were only observed in Arp3‐KD CTLs and not in control CTLs (Figure 5a, control with no bleb). Bleb expansion and retraction dynamics were calculated for more than 600 blebs (about 50/cell) by tracking the bleb masks. Bleb expansion occurred over a 19‐s time span and was faster than retraction, which occurred over a 32‐s time span (Figure 5b, c). Approximately five blebs (5 ± 2) were detected per leading edge of each cell, each comprising ∼ 2% of the total cell area with an average diameter of roughly 3 μm (Figure 5e–g, Supplementary table 1). In addition, blebs were always observed at the leading edge of the CTLs, where expansive forces generated by the contractility of actomyosin cortex are transmitted by cytosolic hydrostatic pressure to the leading edge of the cell (Figure 5h, i).

### Reduction in Arp3 expression levels weakens the actin cortex

**Figure 6:**
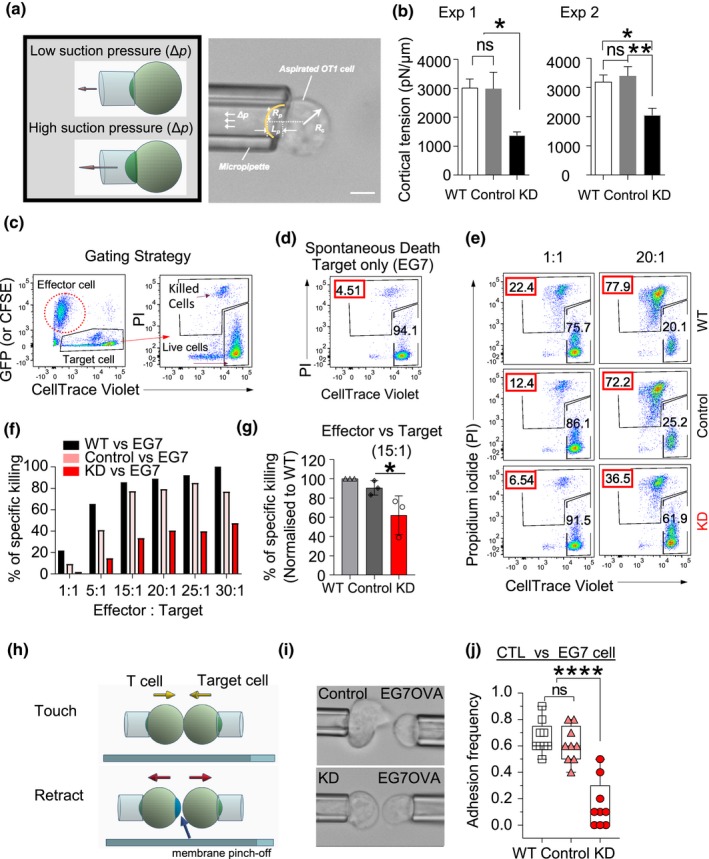
The influence of Arp3 reduction on cytotoxic effector T lymphocyte (CTL) cortical tension, cytotoxic function and interactions with the target cell. **(a)** Schematic diagrams and photomicrographs of micropipette aspiration of a CTL. The aspiration pressure inside the pipette (Δ*p,* low for holding cells, high for cortical tension measurement), the inner radius of the pipette (*R*_p_), the radius of the spherical portion of the cell outside the pipette (*R*_c_) and the length of the cell tongue aspirated inside the pipette (Lp) are indicated. **(b)** The graph shows cortical tensions in OT‐I cells on days 5–6 post‐harvest. Data represent mean ± s.e.m. of two pooled independent experiments [wild type (WT) and vector *n* = 10 and KD *n* = 11 cells/experiment, one‐way ANOVA, followed by Tukey's multiple comparisons test was performed, *P = 0.0148 (exp1), *P = 0.0144 (exp2) and **P = 0.0049] scale bar, 5 μm. **(c)** FACS plot shows the gating strategy for sorted CTLs co‐incubated with the specific (EG7‐OVA) target cells over 3 h measuring the experimental death (killed cells gate). **(d)** The “spontaneous death” of EG7 propidium iodide (PI)^+^ (4.51%) cells, when target cells were incubated alone. **(e)** A representative example of the Arp3‐KD cells co‐incubated with target cells (EG7‐OVA) at 1:1 and 20:1 E/T ratios (WT:target cells, top panel; control:target cells, middle panel, KD:target cells, bottom panel). Red box, the percentage of experimental death. **(f)** Bar graph of a representative cytotoxicity experiment (*n* = 3). See also Supplementary figure 1. **(g)** The column graph shows the percentage of killing normalized to WT for 15:1 E/T ratios of *n* = 3 independent experiments; mean ± s.d., an unpaired Student's *t*‐test was used, *P = 0.03. Cells were FACS sorted on days 6–7 and used in assay on days 9–11 post‐harvest. **(h)** Schematic of single‐cell micropipette adhesion frequency assay. OT‐I T and EG7‐OVA cells were aspirated by two opposing micropipettes, where the target EG7 cell was driven by a piezoelectric translator to touch the T cell for 20 s and then retracted. **(i**, top panel**)** Photomicrograph of a control cell reaching out to a target cell even before the touching step. **(i**, bottom panel**)** A KD cell fails to make any visible interaction, no interaction, and well‐defined shape even during the “touch” step. **(j)** Adhesion frequency measurements for individual T cell–EG7 pairs with 20 s contact durations. Data were pooled from two independent experiments (*n* = 9, four or five cells/experiment average of ten touches/cell). The results are represented in box and Whisker plots, with a median, first and third quartiles outlined by the box, and minimum and maximum values of the data set denoted by Whiskers measurements, ****P < 0.0001 using one‐way ANOVA, followed by Tukey's multiple comparisons test. ns denotes not significant. FACS, fluorescence‐activated cell sorting.

The formation of blebs indicated potential defects in the membrane attachment to the cortical network in Arp3‐KD CTLs. Connections between the membrane and the cortical actin network define the cortical rigidity.^45^ Therefore, to measure the stiffness of the actin cortex which provides support for the plasma membrane, we performed micropipette aspiration experiments. This is a well‐established mechanical measurement technique that assesses membrane‐to‐cortex attachment in an individual cell.^46^, ^47^, ^48^ Individual CTL cortical tension was quantified based on the portion of the cell aspirated into the pipette (Figure 6a). Compared with control CTLs, knockdown of Arp3 resulted in a significant reduction in cortical tension (Figure 6b), suggesting that the actin cortex network and potentially its connection to the membrane was compromised.

### Reduction in Arp3 expression levels impairs cytotoxic T cell function and cell‐to‐cell interactions

Following successful interstitial migration, cytotoxic T cells need to physically contact target cells, which can lead to the formation of an “immunological synapse” and direct the killing of target cells.^49^, ^50^ To evaluate the effect of Arp3 knockdown on cytotoxic T cell function, we used a combination of bulk killing and single CTL:target cell interaction assays over prolonged and short periods of time, respectively. We co‐incubated the Arp3‐KD and control CTLs with cognate (EG7‐OVA) and non‐cognate (EL4) target cells for 3 h. Arp3‐KD CTLs induced a consistent decrease in EG7 target cell lysis compared with control CTLs (Figure 6c–g, Supplementary figure 1e), indicating that cytotoxicity was impaired in Arp3‐KD CTLs. Supplementary figure 3 shows the surface expression of the TCR or activation markers, including CD44, CD25 and CD69, in a set of activated CTLs on day 5 post‐harvest.

To further examine the Arp3‐KD cytotoxicity impairment, we performed single‐cell micropipette adhesion frequency assays to assess the ability of OT‐I CTLs to make contact with their target cells. This technique is frequently used to demonstrate the binding specificity of receptors for their ligands during single cell–cell interactions^46^, ^51^, ^52^, ^53^ wherein adhesion is measured using a binary scoring system (one if adhesion is observed, zero if not). In this assay, multiple CTL–target cell pairs were tested while each pair was evaluated by bringing the cells together ten times with a 20‐s interaction time (Figure 6h, i). We observed a significant decrease in the adhesion frequency of Arp3‐KD CTLs to EG7 cells when compared with control and non‐transduced cells (Figure 6j). Together, these data indicate that disruption of the actomyosin cortex leads to decreased adhesion between CTL and target cell and impaired cytotoxic function of T lymphocytes.

## Discussion

Migration is integral to T lymphocyte function. Indeed, T cells can be considered professional migratory cells that continuously recirculate between the bloodstream and solid organs. In particular, effector T cells are required to quickly adapt to the ever‐changing microenvironments in peripheral tissues during infectious, inflammatory or malignant diseases. Thus, it is not surprising that T lymphocytes employ a highly sophisticated and precisely regulated migration machinery provided by the actomyosin skeleton. While the intricacies of this machinery have been studied in great detail *in vitro*, less is known about the role of Arp3 subunits of the Arp2/3 complex in primary T cells. In this study, we provide evidence that disturbance of the actin nucleator Arp2/3 has remarkable effects on the migration mode of T cells, namely, a switch from the ameboid to a less efficient migratory mode accompanied by blebbing primarily generated at the leading edge. In addition, we demonstrate a role for Arp2/3 in CTL survival and cytotoxicity. These findings underscore the importance of fine‐tuning of the actin nucleators such as Arp2/3 on the actin filament assembly or length in T cell mechanobiology.

In a previous study, a critical role for Arp2/3 in T cell survival has been inferred from ARPC2‐deficient mice.^25^ ARPC2 subunit stabilizes the Arp2/3 complex, and ARPC2 deficiency led to reduced numbers of peripheral T cells without significantly affecting thymocyte development.^25^ Consistently, we also observed a survival defect in Arp3‐depleted CTLs following their activation *in vitro* and adoptive transfer *in vivo*. However, in contrast to complete ARPC2 knockout, we did not observe changes in TCR expression, T cell activation or differences in proliferation kinetics of Arp3‐knockdown CTLs. This may be explained by the modulation of different molecules in the two studies. Alternatively, Arp3 levels in our study were reduced by approximately 60%, which may still allow for unaltered intracellular TCR trafficking. Although mechanistic insight into the decreased survival of Arp3 knockdown warrants further investigation such as using late apoptosis markers, it is conceivable that these cells are less resistant to cellular stress, for example, suboptimal cytokine availability, shear forces in the bloodstream, interstitial fluid pressure or cytothripsis encountered particularly *in vivo*. Cytothripsis (or catastrophic cell death) was observed in DOCK8‐deficient T and natural killer cells during migration in collagen‐dense tissues because of lack of coordination of the cytoskeletal structures.^54^ Future studies will investigate these possibilities in more detail.

Our experiments identify and explore the critical role of Arp3 in the migration of primary effector T cells. Our work is consistent with previous evidence indicating that perinuclear Arp2/3 mediates fast‐moving leukocyte migration through complex 3D environments via nuclear deformation and lamellipodia formation.^55^, ^56^, ^57^ We also found that the exploratory behavior of Arp3‐KD CTLs was adversely affected, which may explain the slight alteration in trafficking patterns of Arp3‐KD T cells into peripheral tissues *in vivo*. T cells can also migrate in an integrin‐dependent manner particularly in nonlymphoid tissues; for example, blockage of integrin (β1) impaired the migration of effector CD4^+^ T cell in the pancreas.^58^, ^59^ Nevertheless, we showed in this study that homing, which also requires integrins to function, is not affected by Arp3 knockdown in liver, lung, spleen or lymph nodes. Previous studies also showed that cancer cells can evade natural killer T‐like and natural killer cells cytotoxic attack by affecting the production of granzyme B.^60^ We have previously shown that granzyme B promotes CTL transmigration,^61^ thus the production of granzyme and perforin in Arp3‐KD cells with the transmigration would warrant further investigation. Moreover, the association of integrin‐dependent migration and the function of Arp2/3 on motility properties of CTLs remains to be determined.

Many studies have shown that blebbing at the leading edge and defects in migratory behavior occurs when cell contractility regulators are manipulated. For example, contractility was augmented as a result of increase in local concentration of myosin light‐chain kinase at the leading edge of Walker carcinoma cells,^62^ fibroblasts and *Dictyostelium*.^33^, ^63^ It is also affected by Rho‐kinase localization at the rear of acute T lymphoblastic leukemia cells^64^ or A375 melanoma cell line.^65^ In another study, cell contractility and cortex integrity were manipulated by knocking down Septin in murine CD4^+^ T helper cells. This increased the transmigration and chemotaxis capacity and reduced the migration speed.^8^ In our study, we report that reduction in Arp3 levels in primary cytotoxic T cells triggers formation of bleb protrusions. This was in contrast to previous studies that reported filopodial (spike‐like) protrusions in the absence of branched actin generated by the Arp2/3 complex in fibroblasts.^66^ We only occasionally detected filopodial features on the periphery of Arp3‐KD CTLs (data not shown). Although the morphology alteration was expected in Arp3‐KD cells, the formation of blebs was surprising because T cells motility in extracellular matrices almost exclusively exhibit lamellipodia/filopodia protrusion locomotion mode.^35^

Blebbing has been suggested to be a more efficient means of exploring the extracellular environment than directional migration, at least in zebrafish mesendoderm cells.^29^ In contrast to lamellipodia, blebs are generally considered less energetically costly,^28^ such that a switch to this mode may allow cancer cells to optimize their migration in different environments^67^, ^68^ and escape antitumor treatments.^69^ Indeed, blebbing migration has been proposed to be a major mechanism by which cancer cells enhance their invasive capability.^70^, ^71^ In this study, however, blebbing CTLs illustrated a restricted exploratory behavior suggesting that Arp2/3 complex‐dependent lamellipodium is a more efficient means of directional migration for T cells. The central role of Arp2/3 is further exemplified by the involvement of all seven subunits of Arp2/3 in cortical actin regrowth and retraction of laser‐induced blebs in HeLa cells in which rapid reduction of actin was observed in the presence of Arp2/3 inhibitor CK‐666.^72^ The biphasic profile of bleb expansion and retraction in cancer cells^47^, ^73^ is similarly shown in CTLs in this study (Figure 6c). Small‐molecule Arp2/3 inhibitors such as CK‐666 and CK‐869 have been used to study the effect of Arp2/3 on actin.^34^, ^71^, ^72^, ^74^ However, their precise effect and their mechanism of action are still under investigation.^74^ Further comparisons between these molecules, along with shRNA and complete knockdown techniques, will shed more light on the influence of individual subunits of Arp2/3 on the actin filament network and their role in transition of blebbing into movement.

The role of cortex tension seems to be particularly important in 3D environments. Previous work by Chugh *et al*. reported that cortex tension is myosin‐II driven.^9^ Here we revealed that uncompromised Arp2/3 complex also plays an important role in retaining and supporting the cellular cortical network and potentially in the membrane‐to‐cortex attachment in CTLs. The observed reduction of membrane tension could be a result of shorter filaments in the absence of a fully functional Arp2/3 complex. Nevertheless, our finding is thus in line with the notion that actin filament length is as important as myosin activity in cell‐shape integrity.^9^ In addition, the strength of membrane to cortex attachment has also been proposed to be important in cellular shape, motility and function^47^, ^75^ (reviewed in Hochmuth^48^). Together, these observations are intriguing as even a moderate reduction of Arp3 leads to a significant disturbance of the membrane integrity. Our results thus suggest that the shape and function of the fast‐moving CTLs are highly affected by a precise equilibrium of actin nucleators and actin filaments, which coordinate the regulation of cell cortical tension, actin network to membrane attachment and downstream functions including migration and cytotoxicity. Although we have observed the same motility properties in our zebrafish model, the reduction of cortical tension might be advantageous for migrating in high‐density microenvironment such as the tumor cores.

Finally, many functions in the cell are dictated by changes in cellular morphology.^76^ Herein we also found that the cytotoxicity function and CTL‐to‐target cell interaction, as representative of initial steps in IS formation, were compromised in Arp3‐KD CTLs. Although both phenomena are Arp2/3 complex dependent and other actin nucleation‐promoting factors such as WAVE2, Wiskott–Aldrich syndrome protein and HS1 have been reported to be important,^77^, ^78^, ^79^ Arp3's role in synapse formation and target cell lysis in primary mouse CTLs were still obscure. Using single cell‐to‐cell interaction assays, we now directly show that reduction in Arp2/3 levels led to diminished membrane interactions with tumor target cells expressing cognate antigen. Notwithstanding, the impaired cytotoxic function of the Arp3‐KD CD8^+^ T cells could also arise from the potential role of the Arp2/3 complex in many more steps, including early T cell death upon target cell encounter, differentiation into effector memory T cells during the *in vitro* stimulation, the strength of TCR activation and/or polarization/docking of lytic granules. Nevertheless, our result is consistent with the recent study by Brigida *et al*., which concluded that ARPC1B is also crucial for the Arp2/3 assembly and maintenance. They showed that alteration in the protein structure of ARPC1B hinders IS assembly.^80^ Together with our observations in bulk cytotoxicity assays, we propose that certain CTL functions as well as their adhesion to the target cells are facilitated by Arp2/3‐dependent cell morphology. The formation of the IS could also be impaired, for example, as a result of the regulation of intracellular trafficking of organelles toward the immunological synapse. Approaches such as confocal imaging or rescue assays, which particularly enhance the TCR‐driven signaling machinery, can be used to further investigate this aspect.^80^

In summary, our study established that the type of protrusion formed by CTLs is fine‐tuned and optimized by the Arp2/3 complex. We demonstrate that the Arp2/3 complex's role is not redundant among the various mechanochemical coordinators involved in the leading‐edge formation in CTLs. Given the importance of the regulation of the actin network in T cell function, we hypothesize that the reported malfunctions in CTLs are mainly a result of the absence of a fully functional Arp2/3 complex and thus shorter actin filaments. These unforeseen findings pave the way for a deeper understanding of the contribution of the Arp2/3 complex to efficient T‐cell migration and function, which is crucial for the development of improved therapies for cancer and inflammatory diseases.

## Methods

### Mice and reagents

Donor recombination‐activating gene 1 (*Rag1*)^−/−^ or OT‐I×GFP‐Lifeact mice were maintained on the C57BL/6 background and bred in‐house at the Centenary Institute. GFP‐Lifeact mice were kindly provided by Roland Wedlich‐Söldner, University of Münster. Congenic C57BL/6‐*Ptprc* ^*a*^ (CD45.1^+^) mice were obtained from the Australian BioResources facility (NSW, Australia). All experiments involving animals were conducted according to animal ethics protocols approved by the Sydney Local Health District Animal Welfare Committee (Sydney, Australia).

### Cell culture

Mouse lymphoma cell line EL4 and its SIINFEKL peptide‐expressing derivative EG7‐OVA were obtained from American Type Culture Collection (Manassas, VA, USA). The cells were maintained at 37°C in a humidified atmosphere of 5% CO_2_. The cells were cultured in complete T‐cell media (TCM) consisting of Roswell Park Memorial Institute Medium‐1640 supplemented with 10% fetal bovine serum, 1 mm sodium pyruvate, 10 mm 4‐(2‐hydroxyethyl)‐1‐piperazineethanesulfonic acid, 100 U mL^−1^ penicillin, 100 μg mL^−1^ streptomycin and 50 μm β2‐mercaptoethanol (Gibco, Thermo Fisher Scientific, Waltham, MA, USA).

### *In vitro* differentiation of primary murine T cells

Splenocytes were isolated from 8‐ to 14‐week‐old (*RAG1*)^−/−^ ×OT‐I or Lifeact‐GFP × OT‐I mice. OT‐I T cells are specific for the OVA_257–264_ peptide (SIINFEKL) in an H2‐K^b^ major histocompatibility complex class I context. Isolated splenocytes were incubated with SIINFEKL peptide (1 μg mL^−1^, Sigma, St Louis, MO, USA) for 2 h at 37°C, in the complete TCM. After 2 h, cells were washed and incubated in TCM containing 10 ng mL^−1^ recombinant mouse interleukin‐2 (R&D Systems, Minneapolis, MN, USA). The medium was changed every alternate day and fresh cytokines were added. Cells were used between days 5 and 10 post‐harvest or 2 days post‐thaw (frozen down on day 4 post‐harvest) or as specified in figure captions.

### Plasmid constructs

mCherry was amplified using ada233 and ada234 primers (ada233: tgtccacaACCATGGTGAGCAAGG, ada234: cgcgttaattaaCTACTTGTACAGCTCGTCC) and cloned into the *Bst*XI and *Pac*I sites of pLMP to replace GFP. For retroviral transduction, mouse ACTR3‐target shRNA (5′‐TGCTGTTGACAGTGAGCGCGCAGATGTAGAAGAGAGCTAATAGTGAAGCCACAGATGTATTAGCTCTCTTCTACATCTGCATGCCTACTGCCTCGGA‐3′) and nonsilencing shRNA (control) encoding sequences^81^ were cloned into the pLMP‐puro‐mCherry vectors. These novel constructs were used to produce ACTR3‐silencing retrovirus and control retrovirus upon introduction into packaging cells.^81^

### Cell transfection and retroviral transduction of primary CD8 effector T cells

Plat‐E packaging cells were transfected with pLMP‐puro‐mCherry constructs encoding control shRNA or shRNA against ACTR3 following manufacturer's instructions.^82^ Transfection was performed using either FuGENE 6 (Promega, Madison, WY, USA) protocol or calcium phosphate method^83^ (or consecutively). The supernatants containing the viral particles were harvested and filtered after 48 and 72 h post‐transfection. The viral supernatants were either immediately used or cryogenically stored; 24 h post‐priming with SIINFEKL peptide, T cells were transduced with viral particles as described previously.^84^ In brief, the retroviral supernatant was co‐incubated with 1.5 × 10^6^ cells mL^−1^ in TCM without antibiotics using nontissue culture plates coated with RetroNectin (15–20 μg mL^−1^). Spinoculation was performed two times in which the plates were centrifuged at 2000*g* at 30°C before incubating them in a cell culture incubator overnight. Transduction efficiency was assessed by the expression of mCherry using flow cytometry on day 5 post‐isolation.

### Flow cytometry

Expression of activation‐associated surface molecules on OT‐I CTLs was evaluated on day 3 or 4 post‐transduction or the day that adoptive transfer was performed, which was usually day 6 post‐isolation. Cells were stained with anti‐CD8α (53–6.7, BD Biosciences, San Jose, CA, USA), anti‐CD44 (IM7, BD Biosciences), anti‐CD25 (PC61, BD Biosciences), anti‐CD69 (H1.2F3, BD Biosciences), anti‐CD62L (MEL‐14, eBioscience, Vienna, Austria) and anti‐V_α2_ (B20.1, BD Biosciences). Antibodies were used at 1 μg mL^−1^ in running buffer (5% fetal bovine serum and 2 mm ethylenediaminetetraacetic acid, 0.01% sodium azide in 1 × phosphate‐buffered saline) and the cells were stained for 20 min at 4°C prior to washing two times with running buffer. Cell viability was evaluated using 0.5 μg mL^−1^ 4,6‐diamidino‐2‐phenylindole (Thermo Fisher Scientific) or the LIVE/DEAD Fixable near‐IR (Thermo Fisher Scientific). Data were collected on an LSR Fortessa flow cytometer (BD Biosciences) and analyzed using FlowJo software (TreeStar Inc., Ashland, OR, USA). For fluorescence‐activated cell sorting of medium to high mCherry expressing CTL population, cells were resuspended in FACS buffer (5% fetal calf serum, 2 mm ethylenediaminetetraacetic acid in 1 × phosphate‐buffered saline) and incubated with 4,6‐diamidino‐2‐phenylindole. Cells were either used fresh or cryopreserved according to established methods.^85^

### Western blot analysis

Transduced OT‐I CTLs were sorted either on day 5 or 10 post‐harvest or immediately post‐thaw (frozen down on day 4 post‐harvest). Sorted mCherry‐positive and mCherry‐negative populations of both Arp3‐KD and control OT‐I CTLs were lysed in cold radioimmunoprecipitation assay lysis buffer containing 1:100 dilution of protease inhibitor cocktail and separated on a 4–12% Tris–glycine gel by equally loading 10–19 μg protein lysate per well/experiment. After protein transfer, each polyvinylidene difluoride membrane was probed with primary rabbit antimouse ACTR3 antibody (Sigma) and the secondary antibody goat antirabbit conjugated to Alexa Fluor 594 (Thermo Fisher Scientific) for protein detection. The primary and secondary antibodies were used at 1:5000 and 1:10000 dilutions, respectively. The stained membranes were imaged with a ChemiDoc MP Gel Imaging System (Bio‐Rad, Hercules, CA, USA) and quantitatively analyzed using ImageJ (NIH) freeware.

### Total F‐actin flow cytometry‐based measurement

Total F‐actin content in transduced and non‐transduced OT‐I CTLs was measured using flow cytometry. Transduced OT‐I CTLs, on day 2 post‐sort (day 7 post‐harvest), were washed with Hank's Balanced salt solution at 3 × 10^5^ cells mL^−1^ concentration and stained with 20 μm phalloidin (conjugated to Alexa Fluor 647 dye; Cell Signaling Technology, Danvers, MA, USA) during 20 min fixation (500 μL of 4% paraformaldehyde, and 0.5% saponin in 1 × phosphate‐buffered saline) at 37°C.

### Annexin V apoptosis detection assay

Early apoptotic states of transduced T cells were evaluated using the Alexa Fluor 647 conjugated Annexin V (Thermo Fisher Scientific). In brief, a total of 1 × 10^6^ cells were incubated in 100 μL of annexin‐binding buffer (10 mm 4‐(2‐hydroxyethyl)‐1‐piperazineethanesulfonic acid, 140 mm NaCl and 2.5 mm CaCl_2_, pH 7.4) and stained with 2 μL of the dye. The cells were diluted in 400 μL of annexin‐binding buffer and analyzed on BD LSR Fortessa after 15 min incubation at room temperature. Cells negative for Annexin V Alexa Fluor 647 nm and 4,6‐diamidino‐2‐phenylindole were considered viable cells. Data were analyzed using FlowJo software.

### CFSE‐ and CellTrace violet‐based proliferation assays

Proliferation assays were conducted as described previously.^86^ In short, freshly isolated, unstimulated splenocytes or transduced T cells (5 × 10^4^ cells) were labeled with 5 μm of carboxyfluorescein diacetate succinimidyl ester dye (CellTrace CFSE, Thermo Fisher Scientific). In experiment one, cells were stained on day 5 post‐harvest and the dilutions of fluorescently labeled live cells were measured by flow cytometry over 3 days post‐staining. In experiment two, cells were stained 2 days post‐thawing on day 8 post‐harvest. In experiment three, we repeated experiment one with 1 μm of CellTrace violet cell proliferation dye (CellTrace CTV, Thermo Fisher Scientific) on days 9–11 post‐harvest. In experiment four, freshly isolated, unstimulated splenocytes were labeled with CFSE. Data were analyzed with the FlowJo software.

### Flow cytometry‐based cytotoxic assay

To evaluate the cytotoxic ability of the transduced OT‐I CTLs, cells were initially FACS sorted either from a freshly isolated transduced OT‐I CTL population or a cryopreserved transduced CTL population (48 h of culture in the cell growth medium is required, day 5 or 6 post‐harvest). Transduced and sorted CTLs derived from either OT‐I or OT‐I×GFP‐Lifeact mice were cocultured with EG7‐OVA or EL4 target cells using fixed numbers of target cells and different effector‐to‐target cell ratios wherein a total of 2 × 10^5^ effector cells were used at a 1:1 ratio. Approximately 10 min before coculturing, transduced OT‐I CTLs and target cells (EG7 and EL4) were labeled with 10 μm CellTrace CFSE and CellTrace violet, respectively. Cells were mixed in 300 μL of TCM and centrifuged at 233*g* for 3 min prior to incubating for 3 h in 5% CO_2_ atmosphere at 37°C. The target cell viability was analyzed using propidium iodide staining (15 nm final concentration) and the specific cytotoxic index (% of specific killing) was calculated using the following formula:

(1) $$\mathrm{Specific}\;\mathrm{cytotoxic}\;\mathrm{index}=\left(\frac{\mathrm{experimental}\;\;\mathrm{death}\;-\;\mathrm{spontaneous}\;\;\mathrm{death}}{\mathrm{maximum}\;\;\mathrm{death}\;-\;\mathrm{spontaneous}\;\;\mathrm{death}}\right)\times 100$$

where “experimental death” is the designated numbers of CellTrace violet‐ and propidium iodide‐positive population at the end of the experiment, the “spontaneous death” is the experimental death obtained from the population of target cells incubated alone and “maximum death” indicates the highest number of deaths detected in control negative (non‐transduced) OT‐I CTL and EG7 cell mixture, respectively. For consistency, a non‐transduced OT‐I CTL population served as the negative control and was subjected to all the experimental conditions similar to control positive and Arp3‐knockdown OT‐I CTL population.

### *In vivo* T cell survival, proliferation and homing assays

T cell survival, proliferation and their ability to traffic into different organs were evaluated using the transduced T cells (7 days post‐isolation) harvested from OT‐I or Lifeact‐GFP×OT‐I mice. A total of 20 × 10^6^ of transduced T cells resuspended in 200 μL of phosphate buffered saline were adoptively transferred via tail vein injection into B6.SJL/*Ptprc* ^a^ (CD45.1) mice. The peripheral blood samples were collected 24 h later and stained with anti‐CD19 (1D3), anti‐CD45.1 (A20), anti‐CD3 (145‐2C11) and anti‐V_α2_ (B20.1), immediately after lysis of red blood cells in ACK lysing buffer (Thermo Fisher Scientific). Subsequently, on day 6 post‐injection mice were euthanized, and blood together with major organs were harvested as described previously.^87^ To obtain single‐cell suspensions, all the tissues were passed through a metal cell strainer (80 μm; Sefar filtration & Metal Mesh, Huntingwood, New South Wales, Australia). Cells were stained with a similar antibody panel as on day 1 and data were collected on an LSR Fortessa and analyzed using FlowJo software. The ratio between injected transduced T cells and harvested T cells was calculated and expressed as the selective homing index (equation (2), 88), and mCherry^+^ normalized to blood (equation (3)):

(2) $$\mathrm{Homing}\;\mathrm{index}\left(\mathrm{HI}\right)=\left(\left[\%\mathrm{mcherr}{y^{+}}_{\mathrm{tissue}}\right]/\left[\%\mathrm{mcherr}{y^{+}}_{\mathrm{input}}\right]\right)$$

(3) $$\mathrm{mCherr}y^{+}\mathrm{normalized}\;\mathrm{to}\;\mathrm{blood}=\left(\left[\%mcherr{y^{+}}_{\mathrm{tissue}}\right]/\left[\%\mathrm{mcherr}{y^{+}}_{\mathrm{blood}(\mathrm{day}6)}\right]\right)$$

### Confocal imaging of T cell migration behavior in 3D collagen

OT‐I CTL migration experiments were performed using either transduced OT‐I or OT‐I× Lifeact‐GFP T cells at days 5–7 post‐isolation (3–5 days post‐transduction) as previously described.^85^, ^89^ In brief, a total of 5 × 10^5^ cells resuspended in phenol red‐free TCM were embedded into neutralized liquid‐phase rat‐tail collagen type I (Corning, NY, USA). The mixture was kept on ice while a volume of 70 μL of a total 100 μL solution was rapidly transferred to a glass chamber. The chamber was made by holding a coverslip in place with vacuum grease on top of a 14 mm glass microwell in a 35 mm petri dish (MatTek, Ashland, MA, USA). To allow the gel to polymerize into a 3D matrix, the cell mixture was incubated for 30 min at 37°C and 5% CO_2_, prior to adding 2 mL phenol red‐free TCM at 37°C to the dish. Cells were imaged 3–4 h post‐incubation. Time‐lapse images were obtained from an approximate 65 μm volume (avoiding 5 μm immediately above the bottom glass coverslip) using a Leica SP5 confocal microscope equipped with a humidified (5% CO_2_) incubator chamber (37°C) and a 20× water immersion objective (1.37 NA, Leica Microsystems, Wetzlar, Germany). The step size was 1.6–1.8 μm every 20–24 s for 20 min while the Lifeact‐GFP and mCherry were excited at 488 and 561 nm wavelengths, respectively. The resulting images were analyzed with Imaris software (Bitplane, Zurich, Switzerland) to obtain individual cell track data and multiple motility parameters including the confinement ratio (track displacement divided by track length) and mean speed of individual cell.

### Time *versus* MSD and straightness Z‐score

To further investigate the exploratory behavior of CTLs, we measured the MSD over time and straightness Z‐score as previously described.^90^ In brief, for each individual track, we calculated displacements for defined time intervals and then calculated the average of all available time intervals. By averaging the MSD of all tracks, we obtained the MSD for the entire population. Based on linear regression, we then computed in 3D, the motility coefficient of analyzed cell populations.

### Confocal imaging of OT‐I CTLs migration behavior in zebrafish model

For *in vivo* study of OT‐I CTL migration, a mixture of transduced and control (non‐transduced) OT‐I CTLs obtained from OT‐I or OT‐ I × Lifeact‐GFP mice were xenotransplanted into zebrafish embryos. Microinjections were performed using standard procedures.^91^ In brief, a total of 60 ± 20 OT‐I CTLs were microinjected into the subcutaneous tissue over the myotome capsule immediately above the venous plexus^92^ of the 20 h post‐fertilization zebrafish embryos. To collect the cell migration motility parameters, imaging of their distances from the injection site was performed at two stages. The first session was conducted 10 min post‐injection to define the injection site in each embryo and the second session was performed approximately 3 h post‐injection during this time embryos were incubated at 37°C and in 5% CO_2_. Transduced CTLs and zebrafish were alive for more than 24 h post‐microinjection in a 37°C incubator. To obtain individual cell mean speed, images were visualized and automatically analyzed using Imaris (Bitplane) with a filter being imposed on cell track analysis to remove smaller than 5 μm track length (to exclude blebbing cells with no movement). To calculate each cell's nearest distance from the injection site, custom code was written in MATLAB (MathWorks, Natick, MA, USA) and the initial injection site was manually assigned. This code is provided in the supplementary data. In brief, the user was prompted to use a slider in a threshold interface to select the cells in each channel by finding the local maxima in the fluorescent intensity. Next, the observer who performed the injection was prompted to locate the injection site in the image. The shortest distance between the cells final position and the injection site was calculated using the following standard equation:

(4) $$\mathrm{Distance}\;\mathrm{from}\;\mathrm{injection}\;\mathrm{site}\left(\mu m\right)=\sqrt{{\left(x_{i}-x_{0}\right)}^{2}+{\left(y_{i}-y_{0}\right)}^{2}}\times \mathrm{Pixel}\mathrm{size}$$

where *x*_*i*_ and *y*_*i*_ are the cell final distance coordinates and *x*_0_ and *y*_0_ represent the coordinates for the injection site.

### Two‐dimensional confocal single‐cell imaging of OT‐I CTLs morphological parameters

The morphological parameters of OT‐I CTLs were examined by evaluating the aspect ratio (maximum/minimum length of the ellipse fitted into the binary mask of cell perimeter) of T cells in confocal images. For this experiment, a mixture of neutralized liquid‐phase rat‐tail collagen type I with phenol red‐free TCM at 1:5 ratios was used to coat the bottom of a 96‐well plates and incubated for 30 min at 37°C and 5% CO_2_. A total of 5000 cells was then seeded on top of the mixture and the plates were subsequently centrifuged at 900*g* for 3 min and incubated at 37°C and 5% CO_2_ for 1 h. Following the incubation, plates were gently transferred into the confocal microscope humidified chamber and imaged using a 10× dry objective (0.4 NA). Alternatively, adhered cells at the bottom of the wells were imaged (with similar results). Cell edge was detected and converted to binary images using MATLAB software. The function “regionprops” was used to measure the length of the major and minor axes of the ellipse fitted into the binary mask.

### Three‐dimensional confocal single‐cell imaging of morphological parameters

Morphological blebbing parameters of T cells were evaluated *in vitro* and *in vivo* by imaging individual cells embedded in collagen type I or injecting them into zebrafish embryos, respectively. Every single cell was observed with a 20× water immersion objective (1.37 NA) on a Leica SP5 confocal microscope at 37°C and 5% CO_2_ with the step size of approximately 0.8 μm every 9–11 s for 3 min (Lifeact‐GFP excited at 488 nm and mCherry at 561 nm wavelengths). Images were visualized and semiautomatically (user prompted to make the adjustment) analyzed using custom code written in MATLAB (MathWorks).

### Quantification of delay between actin cortex and cytoplasm in blebs

The bleb membrane appears devoid of an actin cytoskeleton during expansion and is filled with actin during retraction.^93^ To measure the percentage or mean fluorescence intensity in a bleb during its expansion and retraction, a custom‐written MATLAB algorithm was used and a polygon region of interest was drawn around individual blebs. Time intervals were calculated by dividing the frame time intervals by the number of z‐steps in each z‐stack and every single plane was used as one frame. Subsequently, the average fluorescence intensity within the region of interest for each channel, Lifeact‐GFP and mCherry, was measured at each time point as representative of actin cortex and cell cytoplasm, respectively. Values were exported and the percent fluorescence intensity of each channel at each time point was expressed as a normalized data set between 0 and 1. The number of replicates used for each analysis is specified in the figure captions.

### Quantification of bleb dynamic patterns (position, expansion, retraction and size)

A semiautomated custom‐written MATLAB algorithm was developed to facilitate measuring properties of blebs on T cells. Initially, the maximum intensity projection function was used to compress 3D scenes into 2D images in ImageJ (NIH) software. Then in the MATLAB algorithm, the compressed images were analyzed by prompting the user to adjust the automatically selected region of interest around the whole cell periphery and then manually select a bleb edge using CROIEditor algorithm written by Jonas Reber (http://www.mathworks.com/ matlabcentral/fileexchange/31388‐multi‐roi‐mask‐editorclass/content/CROIEditor/CROIEditor.m). To ensure an accurate bleb region of interest selection, the user was aided by observing the cell with Lifeact‐GFP, mCherry and bright‐field channels simultaneously in ImageJ software while making the decision in MATLAB interface. All the selected features including blebs and whole‐cell boundary were segmented into binary masks and each mask's morphological features were extracted using the “regionprops” function in MATLAB. Given that defining the protrusion/retraction region was a difficult procedure, we used two users to select the region of interest for reproducibility reasons. The feature for each cell includes bleb number per frame, bleb size (percentage of the whole cell area compared with each bleb area) and bleb diameter [length of a major axis (μm) of an elliptical mask created over the binary bleb mask] were measured. Bleb retraction and expansion times were calculated by manually tracking each bleb in consecutive frames and extracting the time intervals between the beginning and the end of expansion and retraction. Bleb positions were also displayed by overimposing the bleb boundary masked on top of each other using “HOT” colormap in MATLAB, where the first and last frames are indicated by white and red, respectively (“+” indicates each bleb center). The number of replicates is reported in the figure captions.

### Measurement of single‐cell cortical tension

The single‐cell cortical tension was measured by the micropipette aspiration method as previously described.^94^ Observations were made with a 60× objective on an Olympus IX70 microscope through a camera (GC1290, Prosilica). In short, at day 2 post‐FACS sorting, transduced OT‐I CTLs were injected into an open‐sided chamber wherein a single cell was held in place with a micropipette. The micropipette aspiration pressure was controlled through a homemade manometer as previously described.^48^, ^51^ Cortical actin layer tension pulls the cell into a spherical shape (with a radius *R*_c_). By fine‐tuning the suction pressure (Δ*p*) of the micropipette, the cell is maintained in a spherical shape while the aspirated region is a hemisphere such that the aspiration length (*L*_p_) approximates the micropipette inner radius (*R*_p_). Therefore, according to the law of Laplace,^48^ the cortical tension (*T*_c_, with units of force per length) can be calculated with the following equation:

(5) $$T_{c}=\frac{R_{c}R_{p}}{2(R_{c}-R_{p})}\Delta p$$

### Micropipette adhesion frequency assay

The capability for a T cell to form a synapse with a target cell (EG7) was measured with the micropipette adhesion frequency assay at room temperature in TCM media as previously described.^46^, ^53^, ^95^ In brief, an OT‐I T cell and an EG7 cell were gently aspirated by two opposing micropipettes, where the EG7 target cell driven by a piezoelectric translator connected to the micropipette was pushed to make a firm touch with the T cell for 20 s and then retracted. Upon retraction, adhesion, if present, was visualized by the T cell membrane pinch‐off. Adhesion frequency is defined as the number of adhesion events divided by the total number of touches (ten touches for each individual T cell–EG7 pair). For each condition, adhesion frequencies ≥ 10 cell pairs in two independent experiments, taking into account the cellular variability, were measured.

### Statistical analysis

Data analysis was mainly performed in Prism software (GraphPad Software Inc, La Jolla, CA). Further statistical details including number of replicates for each experiment are provided in the figure captions.

## Conflict of Interest

The authors declare no conflict of interest.

## Data and Materials Availability

Code used to perform and analyze the data is publicly available at http://tiny.cc/0olrgz. The data discussed in this paper are available upon request from the corresponding author.
