# Supplementary figures and images for: Partial loss of actin nucleator actin‐related protein 2/3 activity triggers blebbing in primary T lymphocytes
Source: Immunol Cell Biol. 2019 Dec 23;98(2):93–113. doi: 10.1111/imcb.12304 (PMC7028084; doi:10.1111/imcb.12304)

Supplementary figure 1

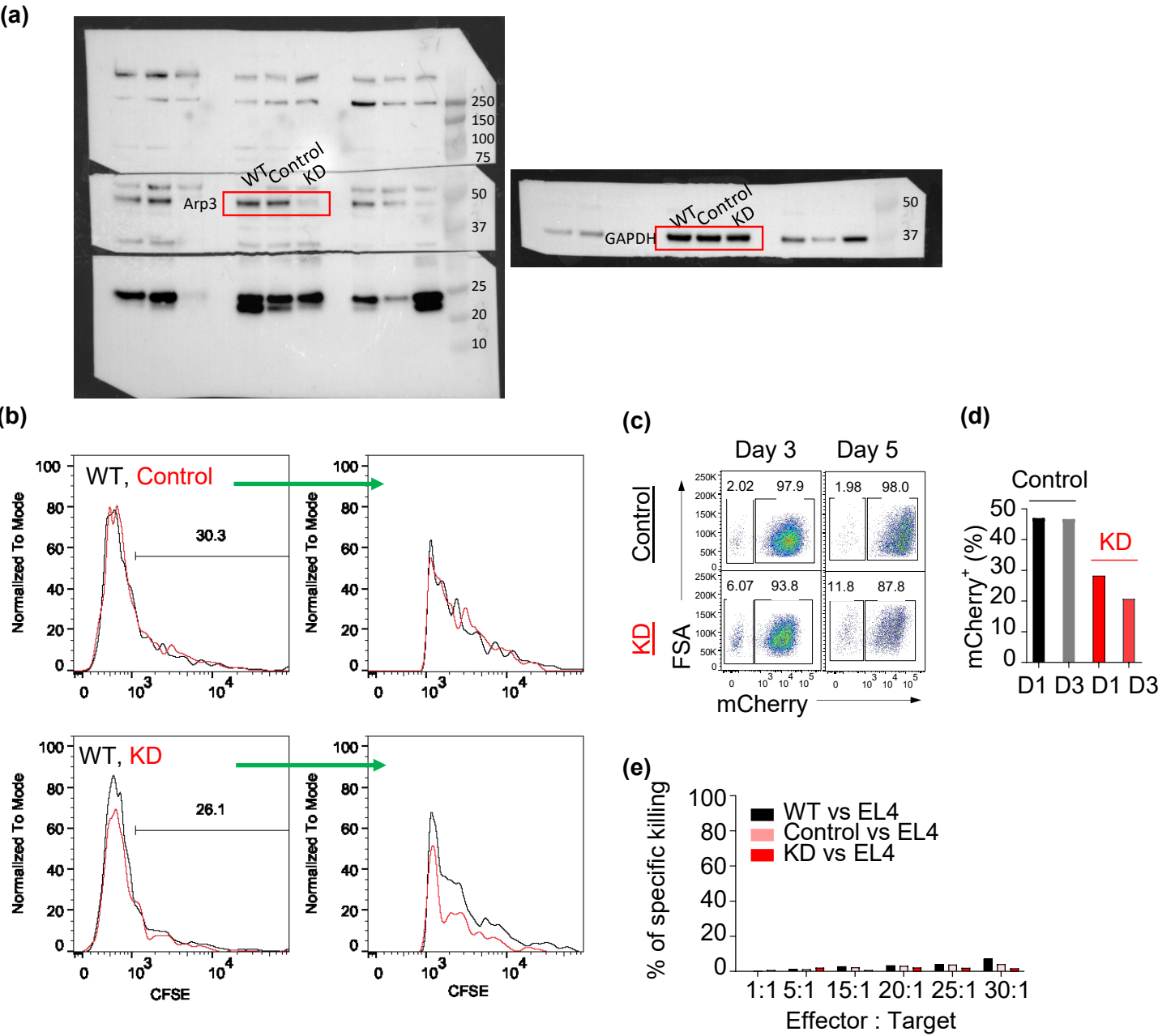

Supplement: Supplementary file 1 [file IMCB-98-93-s001.pdf]

Supplementary figure 2

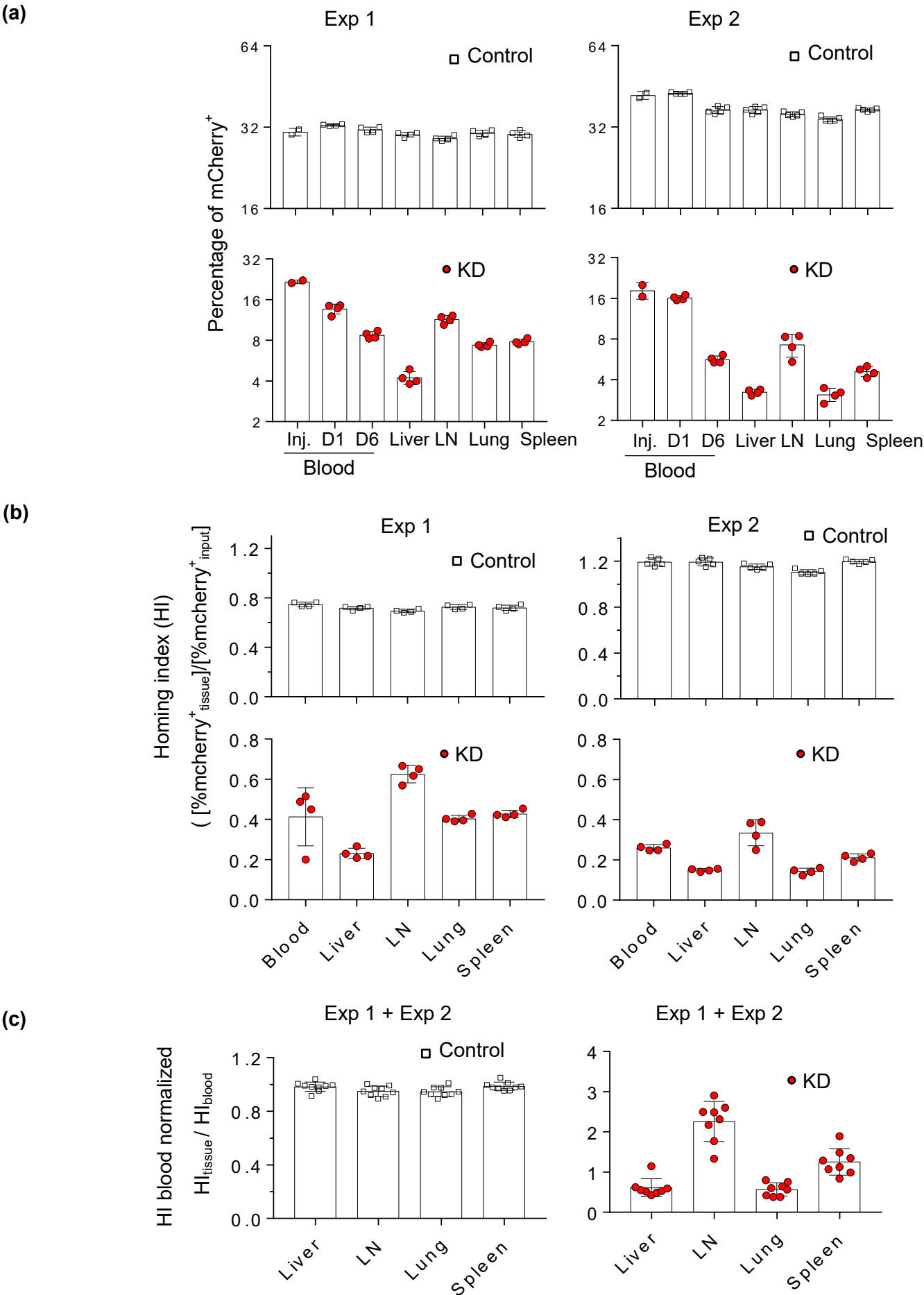

Supplement: Supplementary file 2 [file IMCB-98-93-s002.pdf]

Supplementary figure 3

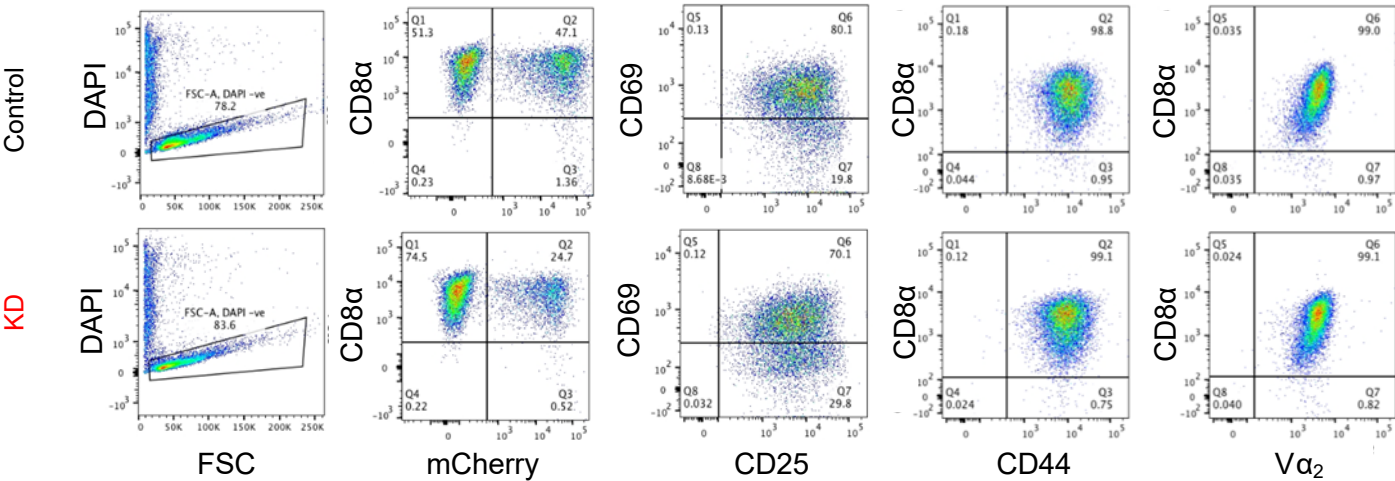

Supplement: Supplementary file 3 [file IMCB-98-93-s003.pdf]
