# Supplementary material for: Partial loss of actin nucleator actin‐related protein 2/3 activity triggers blebbing in primary T lymphocytes
Source: Immunol Cell Biol. 2019 Dec 23;98(2):93–113. doi: 10.1111/imcb.12304 (PMC7028084; doi:10.1111/imcb.12304)
Supplement: Supplementary file 4 [file IMCB-98-93-s004.pdf]

Supplementary table 1

	Bleb number per frame			Bleb size (% of cell size)			Bleb diameter (μm)		
	Exp1	Exp2	Exp3	Exp1	Exp2	Exp3	Exp1	Exp2	Exp3
Median (25%,75% Percentile)	5 (3,6)	4 (4,5)	5 (3,6)	2.1 (0.9,3.7)	1.3 (0.7,2.1)	2.4 (1.3,4.1)	3 (2,4)	2 (2,3)	3 (2,4)
Mean	5	5	5	2.6	1.7	3.2	2.8	2.5	3.5
Std. Deviation	2	1	2	2.4	1.9	2.8	1.4	1.3	1.5
Std. Error of Mean	0.4	0.2	0.2	0.2	0.1	0.2	0.1	0.1	0.1
Number of values	33	39	64	162	179	290	162	179	290
